# Identification of Crucial Amino Acid Residues for Antimicrobial Activity of Angiogenin 4 and Its Modulation of Gut Microbiota in Mice

**DOI:** 10.3389/fmicb.2022.900948

**Published:** 2022-06-06

**Authors:** Mst. Farzana Sultana, Maki Suzuki, Fumiya Yamasaki, Wataru Kubota, Kohta Takahashi, Hirohito Abo, Hiroto Kawashima

**Affiliations:** ^1^Laboratory of Microbiology and Immunology, Graduate School of Pharmaceutical Sciences, Chiba University, Chiba, Japan; ^2^Department of Pharmacy, Jashore University of Science and Technology, Jashore, Bangladesh

**Keywords:** angiogenin 4, antimicrobial peptide, ribonuclease, microbiome, microbiology

## Abstract

Angiogenin 4 bearing ribonuclease activity is an endogenous antimicrobial protein expressed in small and large intestine. However, the crucial amino acid residues responsible for the antibacterial activity of Ang4 and its impact on gut microbiota remain unknown. Here, we report the contribution of critical amino acid residues in the functional regions of Ang4 to its activity against *Salmonella typhimurium* LT2 and the effect of Ang4 on gut microbiota in mice. We found that Ang4 binds *S. typhimurium* LT2 through two consecutive basic amino acid residues, K58 and K59, in the cell-binding segment and disrupts the bacterial membrane integrity at the N-terminal α-helix containing residues K7 and K30, as evidenced by the specific mutations of cationic residues of Ang4. We also found that the RNase activity of Ang4 was not involved in its bactericidal activity, as shown by the H12 mutant, which lacks RNase activity. *In vivo* administration of Ang4 through the mouse rectum and subsequent bacterial 16S rRNA gene sequencing analyses demonstrated that administration of Ang4 not only increased beneficial bacteria such as *Lactobacillus, Akkermansia*, *Dubosiella*, *Coriobacteriaceae* UCG-002, and *Adlercreutzia*, but also decreased certain pathogenic bacteria, including *Alistipes* and *Enterohabdus*, indicating that Ang4 regulates the shape of gut microbiota composition. We conclude that Ang4 kills bacteria by disrupting bacterial membrane integrity through critical basic amino acid residues with different functionalities rather than overall electrostatic interactions and potentially maintains gut microflora *in vivo* under physiological and pathophysiological conditions.

## Introduction

Angiogenin, also known as RNase 5, is a member of the vertebrate-specific ribonuclease A (RNase A) superfamily that was first discovered in a human adenocarcinoma cell line HT-29 and has a wide range of functions, including angiogenesis, cellular processes, tumorigenesis, innate immunity, and neurodegeneration (Fett et al., 1985; Tello-Montoliu et al., 2006; Sheng and Xu, 2016). To date, a number of genes encoding angiogenins in nearly all vertebrates have been identified and are present as a single gene in humans, opossums, and rabbits; two genes in rats, pigs, and turtles; three genes in cattle; five genes in zebrafish; and six genes in mice (Cho et al., 2005; Nitto et al., 2006; Pizzo and D’Alessio, 2007). The sequence similarity within six murine angiogenins is 64–93%, while that with human angiogenin is 55–76% (Crabtree et al., 2007; Iyer et al., 2013). Although the topology of Ang paralogues are highly similar, there is functional diversity (Codõer et al., 2010).

Ang4 is a secreted cationic protein in the gut lumen composed of 120 amino acid residues connected covalently by three disulfide bonds with three α-helices and six β-strands secondary structures with well-documented angiogenic and microbicidal attributes (Pan et al., 2005). Based on structural and biochemical analysis, it was confirmed that Ang4 has three distinct functional sites (Crabtree et al., 2007): (i) catalytic triad comprising H12, K39, and H112 (ii) mammalian cell-nuclear localization sequence comprising three successive residues K30, E31, and R32 and (iii) mammalian cell binding site covering consecutive residues from K58 to N66. Expression of Ang4 in the intestine by paneth and goblet cells during inflammation and *Trichuris muris* infection suggests that Ang4 plays a role in innate immunity (Hooper et al., 2003; Forman et al., 2012). Ang4 exhibits antibacterial activity against *Enterococcus faecalis*, *Listeria monocytogenes*, *Enterococcus gallinarum, Bacteroides thetaiotaomicron, Bifidobacterium longum, and Salmonella typhimurium* (*S. typhimurium*) SL1344 (Hooper et al., 2003; Walker et al., 2013). However, the molecular mechanisms underlying the antibacterial activity of Ang4 remain to be elucidated. Although Ang4 exhibits RNase activity, it is unknown whether this activity is involved in its bactericidal activity. Cationic amino acids (lysine, arginine, and histidine) have been attributed to the antibacterial action of other antimicrobial peptides (AMPs) reported in previous studies (Cutrona et al., 2015; Amirkhanov et al., 2021). In the case of Ang4, mutagenesis studies have been undertaken to determine the residues important for angiogenic activity (Crabtree et al., 2007), but none have been conducted to determine the crucial amino acid residues responsible for Ang4 inducing bacteria killing.

The antimicrobial proteins produced by the epithelial surfaces of the intestine regulate the composition of commensal bacterial communities in the intestinal lumen and protect against pathogen colonization (Mukherjee and Hooper, 2015). However, the *in vivo* regulation of the gut microbiota by Ang4 remains unknown.

Here, we present a mutagenesis study of Ang4 to identify the amino acid residues that play key roles in its antibacterial function. To determine the residues critical for the antibacterial action of Ang4 against *S. typhimurium* LT2, we carried out point mutations of the cationic residue at its functional sites. Our findings show that bacterial membrane permeabilization is required for the killing of bacteria by Ang4, which is accomplished by cationic amino acids present in the cell-binding segment and the N-terminal α-helix of Ang4 structure, revealing mechanistic insights into the antibacterial activity of Ang4. Studies on the impact of Ang4 on gut microbiota have revealed the importance of Ang4 in shaping gut microbiota composition *in vivo*.

## Materials and Methods

### Generation of Recombinant Ang4 and Mutants

The Ang4 gene containing restriction enzyme sequences of *Nco*I and *Hin*dIII with codon optimization for efficient gene expression in *Brevibacillus choshinensis* and oligonucleotides encoding C terminal 6xHis tag attached through a GGGGS linker by GenScript Biotech Co. (Piscataway, NJ, United States). The optimized Ang4 gene was cloned into pNCMO2 which is known as shuttle vector for *Escherichia coli* and *Brevibacillus*. The vector was transformed into *E. coli* DH5α, and after confirming the correct sequence of the target DNA, the vector was introduced into *Brevibacillus* competent cells for protein expression according to the instructions of *Brevibacillus* expression system II (TAKARA). The KOD-Plus-Mutagenesis Kit (TOYOBO) was used to generate mutants of Ang4 using oligonucleotide primers and template DNA (Supplementary Table 1). In brief, inverse PCR of plasmid DNA was performed for 10 cycles using mutation primers. The PCR products were digested with *Dpn*I which cleaves methylated sites of template plasmid. After that, self-ligation of PCR products was performed by T4 Polynucleotide kinase and ligase followed by transformation into *E. coli* DH5α. After confirming the mutations by DNA sequencing, expression plasmids were transformed into *Brevibacillus* competent cells for eliciting protein expression similar to that of wild-type (WT) Ang4.

### Expression and Purification of Wild-Type and Mutants Ang4

For protein expression in the transformed *Brevibacillus* cells, frozen stocks of *B. choshinensis* pNCMO2-WT and mutant Ang4 transformants were incubated in 2SYNm medium at 37°C in water bath shaker (120 rpm) overnight. The culture was diluted to 1:100 ratio in 2SYNm media and again incubated for 64 h at 30°C in water bath shaker (120 rpm). After completion of the incubation period, the supernatant was collected by centrifugation at 7,000×g, 4°C for 15 min. Proteins were precipitated from the culture supernatants using 70% ammonium sulfate saturation (w/v). Precipitated proteins were obtained by centrifugation at 7,000×g, 4°C for 15 min and were further subjected to dialysis in phosphate buffered saline (PBS) to remove ammonium sulfate at 4°C. A His60 Ni Superflow™ Resin & Gravity column (Clontech) was used to purify the tagged recombinant proteins. The dialyzed sample was applied to the column to allow binding of proteins to the resin. After washing, bound proteins were eluted with 300 mM imidazole in 50 mM sodium phosphate and 300 mM NaCl buffer (pH 7.4). To increase protein purity, further purification was conducted using HiTrap™ SP HP cation exchange chromatography (Cytiva) according to the manufacturer’s protocol, in which proteins were eluted using 800 mM NaCl in 50 mM phosphate buffer at pH 7.4. The purity of the Ang4 proteins was evaluated using SDS-PAGE and ImageJ software. Protein concentration was estimated by BCA protein assay kit.

### Colony Count Assay

The antibacterial activity of WT and mutant Ang4 against *S. typhimurium* LT2 was examined by colony count and growth curve assays, as described previously (Doolin et al., 2020; Nazeer et al., 2021b). Briefly, overnight culture of *S. typhimurium* LT2 was diluted in Luria broth (LB) medium (1:100) and incubated at 37°C until the culture reached the log phase of bacterial growth, which was subsequently washed three times with PBS and diluted to 2 × 10^6^ CFU/ml in PBS. Then aliquots of diluted bacterial cells were mixed with different concentration (0.3, 1 and 3 μM) of test proteins, followed by incubation at 37°C for 2 h. After the appropriate dilution, 100 μL of the bacterial suspension were plated onto LB agar plates. Plates were incubated at 37°C for 18 h to assess colony forming units.

### Growth Curve Assay

For growth curve experiments, overnight culture of bacteria was diluted in fresh LB medium and grown to an exponential phase which was subsequently washed three times with PBS and diluted to 2 × 10^6^ CFU/ml in PBS. Then aliquots of diluted bacterial cells were mixed with 1 μM test proteins, followed by incubation at 37°C for 2 h. After incubation, WT- and mutant Ang4-treated bacteria diluted 1:10 in fresh LB medium. Growth curves were generated by measuring absorbance at 600 nm for every 30 min up to 16 h, maintained with continuous shaking at 37°C in 96-well plates (Corning) using a Synergy HTX multi-mode plate reader (BioTek, Tokyo, Japan).

### Determination of Minimum Inhibitory Concentration and IC_50_

Microbroth dilution method was used to determine minimum inhibitory concentration (MIC) and half maximal inhibitory concentration (IC_50_) of Ang4 protein against *S. typhimurium* LT2 as described in the protocol M07-A9 of the Clinical and Laboratory Standards Institute (CLSI, 2015). Different concentrations of Ang4 protein were prepared by 2-fold serial dilution in muller hinton broth (MHB) ranging from 10.64 to 0.04 μM. Overnight culture of *S. typhimurium* LT2 was diluted and incubated at 37°C until the OD 0.5 representing 1∼2 × 10^8^ CFU/mL bacteria. Then the bacterial suspension was diluted with MHB to reach the concentration of 1 × 10^6^ CFU/ml. After that, 100 μL of the bacterial suspension was added to 96 well plate containing 100 μL of serially diluted protein at a final bacterial concentration of 5 × 10^5^ CFU/mL per well. For checking the growth control, same concentration of bacteria with MHB were added to the wells. Then, bacterial growth was observed after 18 h incubation at 37°C by taking absorbance at 600 nm. MIC and IC_50_ of Ang4 protein were determined by GraphPad prism 9 using non-linear regression (curve fit).

### RNA Cleavage Assay

The enzymatic activities of WT and mutant Ang4 toward yeast RNA were assessed by measuring the absorbance of RNA-soluble fragments in perchloric acid at 260 nm, as previously described (Shapiro et al., 1987). Assays containing 2 mg/mL yeast RNA (FUJIFILM), test proteins (0.03, 0.1, 0.3, 1, and 3 μM) and 0.1 mg/mL bovine serum albumin (BSA) in 33 mM Na-Hepes and 33 mM NaCl, pH 7.0 were conducted at 37°C for 2 h. After incubation, the reaction was terminated by chilling on ice and adding two volumes of 3.4% ice-cold perchloric acid. After 10 min on ice, mixtures were centrifuged at 13,200 × g, 4°C for 10 min. Finally, the absorbance of the supernatant at 260 nm was determined as a measure of ribonucleolytic activity.

### Outer Membrane Permeability Assay

The fluorescent dye *N*-phenyl-1-naphthylamine (NPN) uptake assay was used to measure outer membrane permeability, as previously described (Hancock et al., 1991) with some modifications. In this assay, if the outer membrane permeability increases due to the addition of protein, NPN incorporated into the membrane results in enhanced fluorescence. In short, bacteria were incubated with LB medium overnight at 37°C. The overnight culture of bacteria was again diluted to 1:100 in fresh LB medium and incubated at 37°C until it reached a mid-exponential growth phase with an optical density of 0.5, after which it was washed three times with PBS. The bacteria were then diluted to 3 × 10^8^ CFU/mL and proteins were added at a final concentration of 1 μM to the diluted bacteria. An equivalent volume of sterile PBS was used as the control. After 2 h incubation period at 37°C, the samples were incubated for 30 min at 25°C with NPN (Sigma, final concentration 10 μM). Fluorescence was measured at an excitation wavelength of 350 nm and an emission wavelength of 420 nm using a Synergy HTX multi-mode reader.

### Enzyme-Linked Immunosorbent Assay for Binding Assay Against Bacteria

Bacteria were cultured at 37°C to reach exponential phase in LB media. The cells were then washed, and suspended in PBS. High binding, polystyrene, 96-well costar enzyme-linked immunosorbent assay (ELISA) plates (Corning) were pre-coated by incubation with 50 μL of poly *L*-lysine (Sigma, 3 μg/mL) per well in PBS (pH 7.4) for 30 min at 25°C. After the plates were washed three times with 100 μL of PBS per well, 50 μL of bacteria at a concentration of ∼1 × 10^8^ cells in PBS were added to each well. The plates were incubated overnight at 4°C and washed three times with PBS containing 0.05% Tween 20 (PBS-T). Blocking was performed for 1 h at 25°C using 100 μL of 3% BSA-PBS per well to prevent non-specific binding, followed by three washes with 0.05% PBS-T. Fifty microliters of protein at a concentration of 4 μg/mL were added in triplicate to the wells and incubated for 2 h at 25°C. After washing four times with PBS-T, 50 μL of anti-His tag mAb biotin (2 μg/mL, MBL) in PBS-T were added to each well, and the plates were incubated for 1 h at 25°C. The plates were washed five times and 50 μL of streptavidin-HRP (BioLegend) in PBS-T (2,000 times dilution) were added to each well. The plates were incubated at 25°C for 1 h, and then after five times washing, 50 μL of TMB substrate were added to each well. After sufficient color change, the reaction was stopped by the addition of 2 M H_2_SO_4_. Absorbance was measured at a wavelength of 450 nm.

### Flow Cytometry for Propidium Iodide Uptake

To assess bacterial membrane damage, *S. typhimurium* LT2 was grown in LB medium until it reached mid-log phase, then washed three times with 5 mM HEPES and 5 mM glucose buffer, pH 7.4. In the same buffer, the bacteria were diluted to an OD600 of 0.1. Then, 50 μL bacteria were added with 1 μM protein and incubated for 2 h at 37°C. After incubation, propidium iodide (PI) (BioLegend) was added to the mixture at a final concentration of 10 μg/mL and incubated for 30 min in the dark. After staining, bacteria were again diluted to 1:10 ratio in 5 mM HEPES and 5 mM glucose buffer, pH 7.4 and 200 μl of it was added to the 96 well plates. A CytoFLEX flow cytometer (Beckman Coulter) was used to examine the samples and cytometry data analysis was performed by FlowJo software.

### Animals

Female C57BL/6J mice, aged 4 weeks, were purchased from Charles River Laboratories Japan and housed in the animal room of Chiba University for additional 3 weeks for stabilization of microbiota before experiments. All mice were treated in accordance with the guidelines of the Chiba University Animal Care and Use Committee.

### Bacterial Flow Cytometry for Binding Assay

Feces from female C57BL/6J mice were suspended in ice-cold PBS to obtain a 100 mg/mL suspension. Then the sample was centrifuged at 50 rcf, 4°C for 15 min, and the supernatant was passed through 70 μm mesh followed by washing with ice cold PBS to collect bacterial pellet. To prevent non-specific binding, the bacterial pellet was incubated on ice for 30 min in 400 μL PBS containing 3% BSA. After washing with 0.1% BSA in PBS, the bacteria were incubated with Ang4 (10 μg/mL) for 1 h on ice. Bacteria were then washed three times and stained with biotinylated anti-His-tag monoclonal antibody (2 μg/mL, MBL) on ice for 30 min, followed by three washes. The sample was then incubated with streptavidin-PE (0.4 μg/mL, BioLegend) on ice for 30 min, followed by three washes. Finally, the sample was incubated with thiazole orange (0.4 μg/mL, Sigma-Aldrich) to detect bacteria. After washing, the Ang4 binding bacteria were detected using flow cytometry and data analysis was performed by FlowJo software.

### 16S rRNA Gene Sequencing

All sequencing procedures were performed on Illumina MiSeq platforms with primers targeting the variable regions V3 and V4 of the 16S rRNA gene. Briefly, 50 μg of Ang4 in 100 μL PBS were administered rectally to 7-week-old female C57BL/6J mice on days 0, 2, and 4. The same volume of PBS was used for each mouse as a control. After three administrations of Ang4 and PBS, feces were collected on the subsequent day and stored at −80°C until DNA extraction. DNA was isolated from frozen stool samples using the NucleoSpin DNA Stool kit (Takara) in accordance with the manufacturer’s instructions. The V3–V4 regions of the 16S rRNA gene were amplified by PCR and sequenced on a MiSeq platform (Illumina). The raw reads were processed and analyzed using the QIIME 2 software package.^1^ DADA2 was used for forward and reverse sequence merging, quality checking, and amplicon sequence variants (ASV) clustering. Silva 132 sequencing database was used for taxonomic classification. Furthermore, alpha diversity (Shannon entropy) and beta diversity (unweighted UniFrac measures) were calculated to determine the overall microbial community differences between the two groups using the same software packages. To examine the significant differences in bacterial taxa (phyla, classes, families, and genera), the relative abundance was measured for each sample.

## Results

### Generation of Recombinant Ang4 and Its Mutants

**FIGURE 1:**
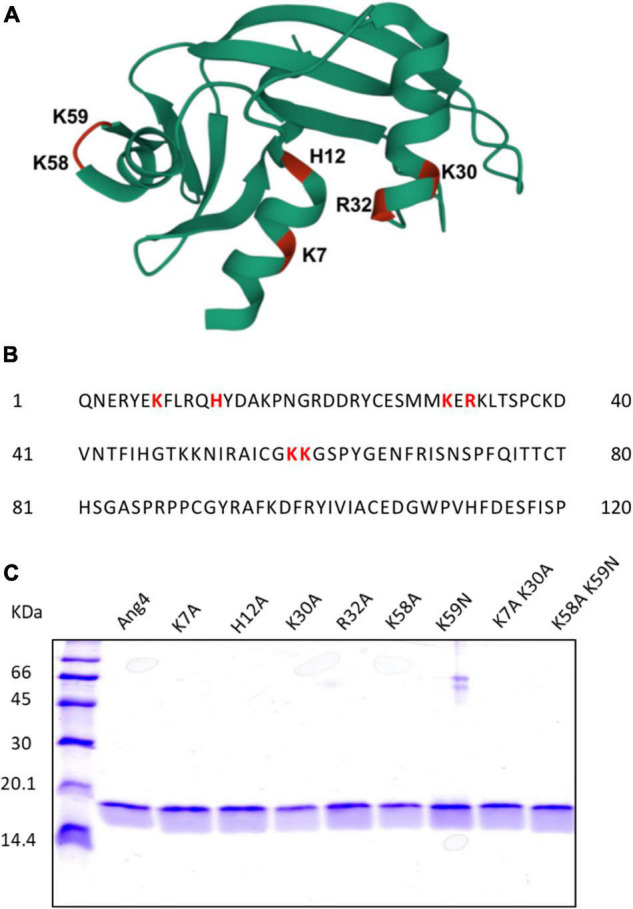
Structural overview and generation of WT and Mutant Angiogenin 4. **(A)** 3D structure of Ang4 (PDB entry 2J4T). The mutated residues in this study are highlighted in color and labeled. **(B)** Amino acid sequence of Ang4. **(C)** SDS-PAGE of purified WT Ang4 and its mutants. Proteins were electrophoresed on a 15% polyacrylamide gel and stained with the Coomassie Brilliant Blue R-250 dye.

The crystal structure of Ang4 (PDB entry 2J4T) contains 120 amino acids with three α-helices and two triple-stranded antiparallel β sheets that contain three functional sites: catalytic site, mammalian cell-nuclear localization sequence, and mammalian cell-binding segment (Figure 1A). In this study, we focused on cationic residues present in different functional sites to examine the contribution of these residues to bacterial killing. In the Ang4 structure, three residues (H12, K39, and H112) are responsible for catalytic activity, and amino acids from K58 to N66 (KKGSPYGEN) act as cell-binding segments (Crabtree et al., 2007). In the N-terminal region, there are two α-helices in which K7 is the center of the first α-helix and seems to be important for the biological activity of Ang4 (Crabtree et al., 2007). Another α-helix contains the mammalian cell nuclear localization sequence (K30, E31, and R32) that is important for transporting the protein into cellular targets (Crabtree et al., 2007). To explore the residues responsible for Ang4’s antibacterial effects, we generated H12A mutants from the catalytic region; K7A, K30A, R32A, and K7A/K30A double mutants from the N-terminal α-helix; and K58A, K59N, and K58A/K59N double mutants from the cell-binding segment. The amino acid sequence of Ang4 is shown in Figure 1B. Recombinant Ang4 and its mutants were expressed in *B. choshinensis* with C-terminal His-tags and purified by His60 Ni Superflow™ Resin and HiTrap™ SP HP cation exchange column chromatography to a purity of >95%, as confirmed by SDS-PAGE (Figure 1C).

### Ang4 Exerts Antibacterial Activity by Disrupting Membrane Integrity

**FIGURE 2:**
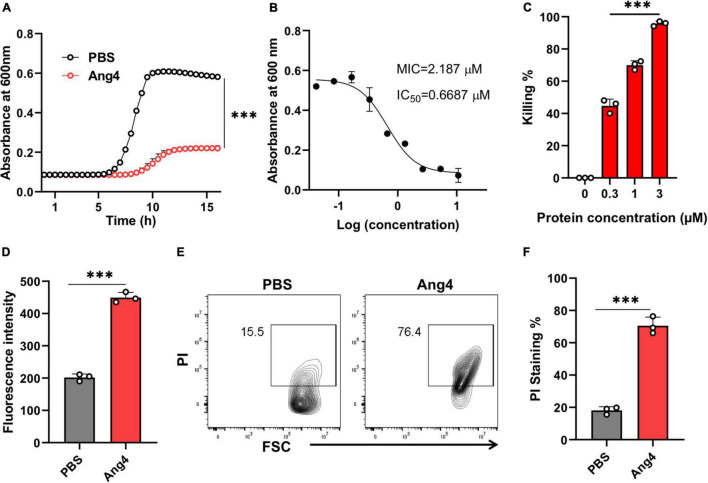
Ang4 disrupts the membrane integrity of *Salmonella typhimurium* LT2. **(A)** Growth curve of bacteria treated with Ang4 measured by OD 600. **(B)** MIC and IC_50_ of WT Ang4 determined by microbroth dilution method. **(C)** Killing percentage of Ang4-treated bacteria after 18 h incubation at 37°C. **(D)** Outer membrane permeabilization by Ang4 was evaluated by measuring the fluorescence intensity of NPN. **(E,F)** Cell membrane alteration in Ang4 treated bacteria was assessed by PI staining using flow cytometry. All data are shown as mean ± SD from three independent experiments. *P*-values were determined by two-tailed unpaired *t*-test or one-way ANOVA; ^***^P < 0.001 vs. control.

We first confirmed the antibacterial activities of the recombinant Ang4 against *S. typhimurium* LT2 using kinetic growth curve assay and killing assay (based on colony forming units). As predicted, Ang4 exhibited potent inhibitory effects on the growth of *S. typhimurium* LT2, as assessed by the growth curve assay (Figure 2A). To determine the concentration that completely inhibits the bacterial growth, MIC was calculated. The results indicated that after incubation of bacteria with various concentration of Ang4 protein, bacterial growth curve decline with increasing protein concentration and the MIC was determined to be 2.187 μM (Figure 2B). In addition, IC_50_ of Ang4 was calculated to be 0.6687 μM, demonstrating the potency of Ang4 (Figure 2B). Then to examine whether this growth inhibition is bactericidal or bacteriostatic, colony count assay was performed after treating bacteria with different concentration of protein. The results showed rapid bacterial reduction, suggesting that treatment with WT Ang4 had bactericidal effects. These data also revealed that killing percentage of Ang4 was increased in dose dependent manner and at 3 μM concentration around 90% bacteria were killed (Figure 2C). Next, we examined the possible mechanism by which Ang4 kills bacteria. Since Ang4 is a cationic protein, it is possible that the cationic residues might bind to anionic bacterial surfaces and subsequently disrupt their membranes. In addition, previous literature suggested that RNase A superfamily proteins have the ability to bind bacteria and subsequently penetrate bacterial phospholipid bilayers (Dyer and Rosenberg, 2006). As expected, the outer membrane integrity, measured by the NPN uptake assay, showed a distinct increase in fluorescence intensity after 2 h of incubation with 1 μM protein (Figure 2D). In addition, PI uptake assay using flow cytometry was performed to assess whether Ang4 proteins target the bacterial membrane. Two hours after Ang4 treatment, approximately 70% of the PI-positive bacteria were detected, indicating that the inner membrane permeability of the bacteria was increased (Figures 2E,F). These results demonstrate that Ang4 exerts antibacterial action by disrupting the integrity of the bacterial membranes.

### RNase Activity Is Not Responsible for Antibacterial Activity

**FIGURE 3:**
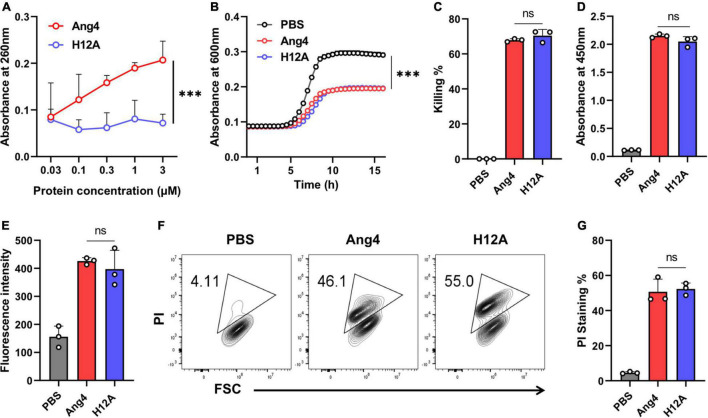
Antimicrobial activity of Ang4 against *Salmonella typhimurium* LT2 is independent of RNase activity. **(A)** RNase activity of Ang4 and H12A determined by measuring the absorbance of perchloric acid soluble RNA fragments after 2 h incubation of assay mixtures at 37°C. **(B)** Growth profile of bacteria incubated with or without Ang4 or H12A at 37°C for 2 h. **(C)** Killing percentage of bacteria that were treated with PBS alone, Ang4, or H12A for 2 h before plating on LB agar plates. **(D)** Binding of Ang4 and H12A to bacteria was evaluated by ELISA. **(E)** The permeability of the bacterial outer membrane induced by Ang4 or H12A was assessed by measuring the fluorescence of NPN. **(F,G)** Flow cytometry to detect the inner membrane permeability of bacteria exposed to Ang4 or H12A using PI. All data are shown as mean ± SD from three independent experiments. P-values were determined by two-tailed unpaired *t*-test or one-way ANOVA with Tukey’s multiple comparisons test; ns > 0.05, ^***^P < 0.001 vs. WT Ang4.

In the Ang4 structure, residues H12, K39, and H112, corresponding to the RNase catalytic triad, were previously reported (Crabtree et al., 2007). The replacement of these residues with non-polar amino acids decreases RNase activity (Crabtree et al., 2007). In this study, the H12A mutant was generated to evaluate the involvement of RNase activity of Ang4 in its antibacterial activity against *S. typhimurium* LT2. First, we confirmed RNase activity of WT Ang4 and H12A mutant at the concentration ranging from 0.03 to 3 μM. The data revealed that the mutation at H12 significantly reduced RNA cleavage activity (Figure 3A), confirming the pivotal role of this residue in catalytic action. The catalytic inactivity of the H12A mutant was consistent with that of the equivalent hANG mutant H13A (Shapiro and Vallee, 1989). We evaluated the contribution of catalytic amino acids to antibacterial activity. We observed that the RNase activity-defective mutant (H12A) had the same antibacterial activity as that of WT Ang4, as assessed by the growth curve and killing assay (Figures 3B,C). In addition, the H12A mutant caused no significant changes in bacterial binding compared to WT Ang4 (Figure 3D). Consistent with the ELISA data, we found that the outer membrane integrity was damaged by WT Ang4 and H12A mutants to a comparable level, which was manifested by similar increase in the fluorescence intensity measured 2 h post incubation (Figure 3E). Moreover, PI uptake assay using flow cytometry was performed to assess whether Ang4 and its mutant target the bacterial inner membrane. Similar levels of PI-positive bacteria were detected after treatment with WT Ang4 and H12A, indicating the comparable increase in the inner membrane permeability of the bacteria (Figures 3F,G). Overall, these findings suggested that RNase activity is not involved in the bactericidal activity of Ang4.

### Cationic Amino Acid Residues in the N-Terminal α-Helix Are Not Essential for Binding but Are Important for Killing

**FIGURE 4:**
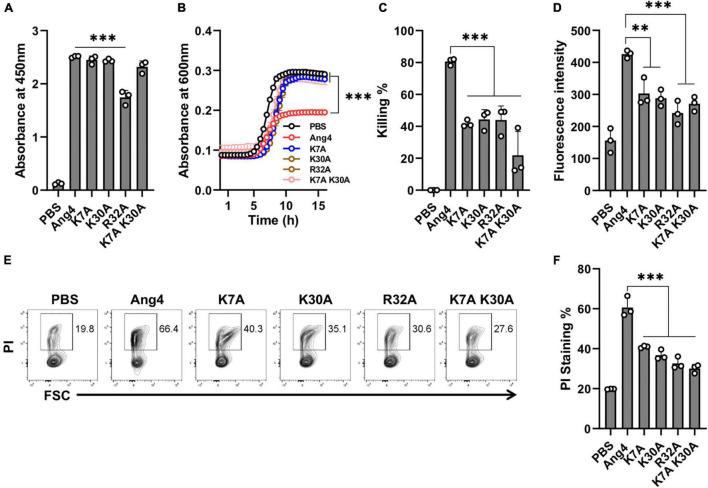
Cationic amino acids at the N-terminal α-helix are not responsible for binding but play a role in killing of bacteria. **(A)** Binding of Ang4 and its mutants to the bacterial cell membrane, as measured by ELISA at 450 nm. PBS was used as a control. **(B)** *Salmonella* growth curve after 2 h of incubation with or without Ang4 or its mutants. **(C)** Bacterial killing assay after 2 h of incubation with Ang4 or its mutants, as measured by colony count. **(D)** NPN uptake assay to identify bacterial outer membrane rupture following addition of Ang4 or its mutants. **(E,F)** PI staining to assess changes in bacterial cell membrane integrity after 2 h of treatment with Ang4 or its mutants. All data are presented as mean ± SD from three independent experiments. P-values were determined by one-way ANOVA with Tukey’s multiple comparisons test; ns > 0.05, ^**^P < 0.01, ^***^P < 0.001 vs. WT Ang4.

In the N-terminal α-helix, three consecutive residues (K30-E31-R32) comprise a crucial element of the mammalian cell nuclear localization sequence (NLS) of Ang4. However, the presence of extra positive residues (Arg10 for Thr11 and Lys7 for His8) in the immediate vicinity of Ang4, compared to hANG, may contribute to a bipartite NLS (Jans et al., 2000; Crabtree et al., 2007). Because bacteria do not have a membrane-enclosed nucleus and DNA is found in the nucleoid floating in the cytoplasm, the contribution of cationic residues present in this sequence, to the function of Ang4 against bacteria is unknown. To determine this function, we generated alanine mutants, including the K7A, K30A, R32A, and K7A/K30A double mutants. The results demonstrated that, apart from R32A, none of these mutants exhibited a significant difference in bacterial cell binding compared with that of WT Ang4 (Figure 4A), indicating the lack of participation of these cationic residues in binding. Interestingly, antibacterial activity estimated by the growth curve and killing assay showed significant changes compared to that of WT Ang4 (Figures 4B,C). These findings suggest that cationic amino acids in the NLS or the N-terminal α-helix region of Ang4 are important for bacterial killing. It has been shown that the killing of bacteria by AMPs is critically dependent on the disruption of cell integrity or cellular targets (Benfield and Henriques, 2020). Consistently, bacterial membrane permeability of these mutants examined by NPN and PI uptake assays revealed less fluorescence intensity and PI-stained bacteria when treated with the mutants compared to WT Ang4 (Figures 4D–F), suggesting the participation of the abovementioned cationic residues in the increase of cell membrane permeability. Taken together, these observations indicate that membrane disruption of bacteria by Ang4 is key for its killing activity and that cationic amino acid residues at the N-terminal α-helix of Ang4 are not essential for its binding to bacteria but are important for its bactericidal activity.

### Cationic Residues in the Mammalian Cell Binding Site Are Critical for Bacterial Binding and Killing

**FIGURE 5:**
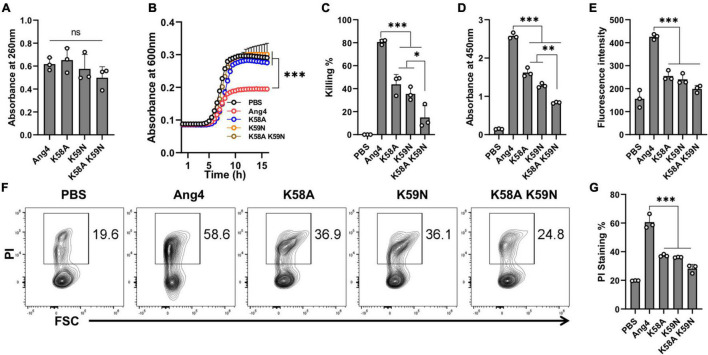
Cationic residues in the cell binding segment of Ang4 are crucial for the bacterial cell binding and killing. **(A)** RNase activity of Ang4 and its mutants was measured by absorbance at 260 nm. **(B)** Growth curve of bacteria after incubation with or without Ang4 or its mutants for 2 h. **(C)** Killing assay of bacteria performed 2 h post-incubation with Ang4 or its mutants estimated by colony count. PBS was used as a control. **(D)** Binding of Ang4 and its mutants to the bacterial cell membrane was estimated by ELISA at 450 nm. **(E)** NPN uptake assay to detect outer membrane disruption of bacteria after the addition of Ang4 or its mutants evaluated by increased fluorescence intensity. **(F,G)** PI staining to evaluate the alteration of bacterial cell membrane after 2 h post incubation at 37°C with Ang4 or its mutants. All data are presented as the mean ± SD from three independent experiments. *P*-values were determined by one-way ANOVA with Tukey’s multiple comparisons test; ns > 0.05, *P < 0.05, ^**^P < 0.01, ^***^P < 0.001 vs. WT Ang4.

The mammalian cell binding site covering the range of residues from K58 to N66 has been implicated to be important for the angiogenic activity of Ang4 (Crabtree et al., 2007). This region (KKGSPYGEN) contained two consecutive cationic amino acids (K58 and K59). However, it remains unknown whether these residues are involved in bacterial binding. To investigate the contribution of these cationic residues, mutations were inserted by substitution of lysine with alanine or asparagine to generate K58A and K59N single mutants as well as the K58A/K59N double mutant. The RNA cleavage assay showed that all mutants had RNA cleaving activity comparable to that of WT Ang4 (Figure 5A), which is consistent with the findings mentioned previously (Hallahan et al., 1991). However, growth inhibition by these mutants was significantly attenuated compared to that by WT Ang4 (Figure 5B). In addition, these mutants showed significantly reduced killing activity than WT Ang4 toward *S. typhimurium* LT2 in the colony count assay (Figure 5C). These data indicate that both K58 and K59 are functionally important for the bactericidal activity of Ang4 against bacteria.

We then examined the possible mechanism underlying the reduced killing of bacteria by these mutants. The binding assay determined by ELISA revealed that the binding of the abovementioned mutants to the bacteria was significantly reduced (Figure 5D). Thus, these mutants may be unable to interact effectively with bacteria because of the replacement of one or more residues that are critical for binding. We then examined the uptake of NPN to determine the influence of these mutations on the permeability of bacterial outer membranes. As expected, NPN uptake by these mutants was significantly lower than that by WT Ang4, as demonstrated by the reduced enhancement of fluorescence (Figure 5E). Similarly, reduced percentage of PI-stained bacteria was observed by flow cytometry after treatment with these mutants (Figures 5F,G). Collectively, these results indicated that K58 and K59 are important for the binding of Ang4 to bacteria, which is required for the increase of the membrane permeability and bacterial cell disruption.

To further clarify the function of the specific amino acids on the binding of Ang4 to bacteria, we also examined the binding of Ang4 and its mutants with mouse fecal bacteria by flow cytometry and ELISA. Both results showed that the binding of WT and mutant Ang4 to fecal bacteria was consistent with their binding to *S. typhimurium* LT2 (Supplementary Figure 1). The binding detected by WT Ang4 was not significantly different from that observed with K7A, H12A, K30A, and K7A/K30A mutants, although the R32A mutant displayed a lower tendency to bind to fecal bacteria. A significant reduction in fecal bacterial binding was observed with K58A, K59N, and K58A/K59N double mutants, consistent with their binding to *S. typhimurium* LT2. These results further confirmed that cationic residues K58 and K59 in the cell-binding segment of Ang4 play a significant role in bacterial binding.

### Overall Structure of the Gut Microbiota Community After Administration of Ang4

**FIGURE 6:**
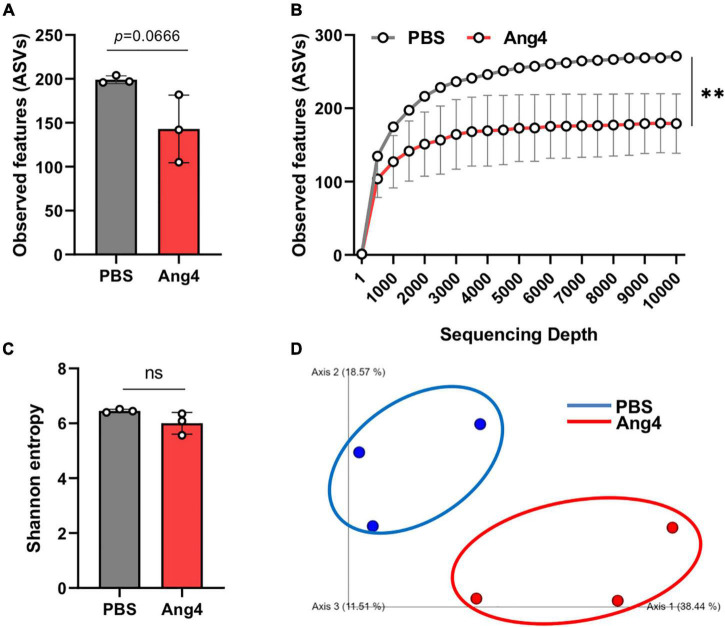
Overall structure of the gut microbiota community after administration of Ang4. **(A)** Observed features (ASVs) determined by Illumina MiSeq platforms in PBS- and Ang4-treated groups. **(B)** Rarefaction curves were calculated at multiple sequence depths. **(C)** Shannon entropy to compare differences in alpha diversity between the two groups. **(D)** Beta-diversity of the gut microbiota using unweighted UniFrac measures. *P*-values were determined using two-tailed unpaired *t*-test or one-way ANOVA with Tukey’s multiple comparisons test; ns > 0.05, ^**^P < 0.01.

Antimicrobial proteins are important for maintaining gut homeostasis and inflammation (Zong et al., 2020). However, the role of Ang4 in shaping the gut microbiota remains unknown. To determine the changes in gut bacteria following administration of Ang4, 16S rRNA gene sequencing was performed. Bacterial DNA samples were isolated from mouse feces after intrarectal administration of Ang4 or PBS three times every other day. Illumina MiSeq sequencing yielded approximately 141 and 199 ASVs, or observed features, for Ang4- and PBS-treated mice, respectively, suggesting that the number of species after administration of Ang4 was diminished (Figure 6A). The alpha rarefaction curve also showed significant reduction in the observed features in Ang4-treated mice with respect to the sequencing depth (Figure 6B). To estimate the overall structure of the microbial community in the Ang4- and PBS-treated mice, alpha and beta diversity measures were calculated. Alpha diversity provides information on microbial diversity within individual samples, and beta diversity assesses the variances between samples. Shannon entropy, an alpha-diversity measurement of richness and evenness, revealed no significant difference between PBS- and Ang4-treated mice (Figure 6C). To determine whether the entire microbial community structure was dissimilar between the two groups, we measured the differences in beta diversity using the unweighted UniFrac distance. Statistical data of the resulting matrices showed that three data points of Ang4-1treated mice were close together but were separated from the data points of the PBS-treated group (Figure 6D).

### Administration of Ang4 Alters Composition of Gut Microbiota

**FIGURE 7:**
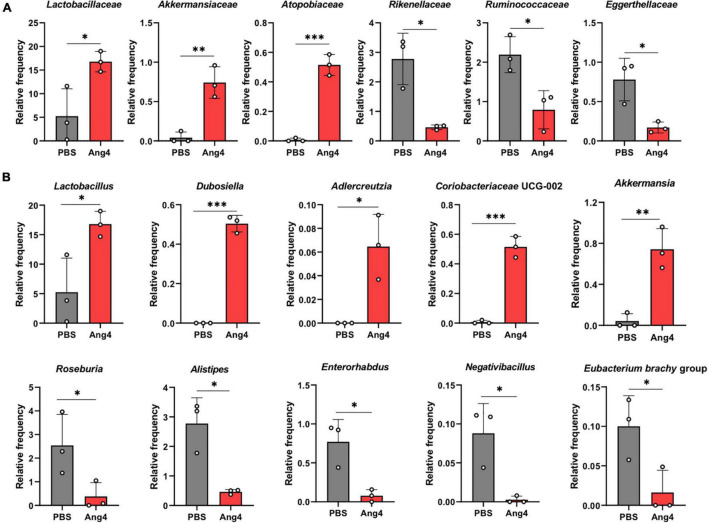
Administration of Ang4 alters composition of gut microbiota. **(A)** Relative abundance of bacteria at the family level. **(B)** Relative abundance of the genera. All figures represent the fecal bacteria that were significantly altered between Ang4- and PBS-treated mice. *P*-values were determined by two-tailed unpaired *t*-test; *P < 0.05, ^**^P < 0.01, ^***^P < 0.001.

To identify the significantly altered gut microbiota between the PBS- and Ang4-treated groups at different taxonomic levels, including phyla, classes, families, and genera, the relative abundance of the microbiota was calculated. At the phylum level, the microbiota of both groups was dominated by *Bacteroidetes* and *Firmicutes*, and to a lesser extent included *Proteobacteria*, *Verrucomicrobiota*, and *Actinobacteriota* (Supplementary Figure 2A). Comparison of the relative abundances of microbiota at the phylum level revealed a significant increase in the phylum *Verrucomicrobia* in Ang4-treated mice compared with that in PBS-treated mice (Supplementary Figure 2B). At the class level, the predominant bacteria were *Bacteroidia*, *Clostridia*, and *Bacilli*, with minor presence of *Verrucomicrobiae*, *Gammaproteobacteria*, *Coriobacteriia*, and *Actinobacteria* (Supplementary Figure 2C). However, a comparison of the relative abundance of bacteria revealed that *Bacilli* and *Verrucomicrobiae* were significantly increased in Ang4-treated mice, whereas *Clostridia* was decreased in those mice (Supplementary Figure 2D). Family level analysis revealed that the relative abundance of *Lactobacillaceae*, *Akkermansiaceae*, and *Atopobiaceae* bacteria was increased, while that of *Rikenellaceae*, *Ruminococcaceae*, and *Eggerthellaceae* bacteria was decreased in Ang4-treated mice (Figure 7A). We further examined whether the relative abundance of bacteria differed between the two groups at the genus level. The results clearly showed specific differences in Ang4-treated mice, including increases in *Lactobacillus, Dubosiella*, *Adlercreutzia*, *Coriobacteriaceae* UCG-002, and *Akkermansia*, and a decrease in *Roseburia*, *Alistipes*, *Enterohabdus*, *Negativibacillus*, and *Eubacterium brachy* group (Figure 7B).

## Discussion

In the present study, we identified crucial cationic amino acid residues with different functionalities in the antibacterial activity of Ang4. Our findings indicate that Ang4 exerts potent antibacterial activity against *S. typhimurium* LT2 *via* membrane permeabilization. The antibacterial activity of Ang4 does not require RNase activity but is dependent on binding to bacteria through the cationic amino acid residues in the cell-binding segment as well as those in the N-terminal α-helix to induce permeabilization. The combination of these two functional sites of Ang4 may act sequentially to kill bacteria. In addition, studies on gut microbiota composition after the administration of Ang4 have revealed its potential roles on maintaining gut microbiota.

Our results demonstrate that the antibacterial activity of Ang4 is not linked to RNase activity. For instance, mutation of H12 by alanine, which is an integral part of its catalytic site, abolished RNase activity but retained antibacterial activity similar to that of the WT. This finding is consistent with other members of the RNase A superfamily, such as RNase 7 and RNase 3, which display antimicrobial activity based on lipid bilayer disruption rather than RNase activity (Huang et al., 2007; Torrent et al., 2010). We found that the cationic residues K7 and K30 in the N-terminal α-helix were critical for killing but not binding to the bacterial surface. However, R32 appeared to be involved in binding to bacterial cells, possibly because this residue is located at the end of the α-helix. The importance of the N-terminal α-helix for antimicrobial activity and membrane destabilization through residues 24–45 and residues 8–16, respectively, has been mentioned by human canonical RNases (Torrent et al., 2009, 2011, 2013). These findings are partially consistent with our data because mutants (K7A, K30A, R32A, and K7A/K30A) demonstrated less permeability than that of WT Ang4. Simultaneously, however, this finding raised a question regarding the critical residues responsible for Ang4 binding to the bacterial surface. Therefore, further mutational studies were conducted. Our data indicate that cationic residues in the cell-binding site, K58 and K59, are critical for both binding and killing activity, imparting a new insight into the bacterial binding site of Ang4.

Our results also demonstrated that significant enhancement of bacterial membrane permeability is pivotal for killing by Ang4. This finding resembled of most AMPs that mediate bacterial killing by disrupting bacterial cell membranes, demonstrated by the increase in fluorescent intensity of NPN and PI, which represents membrane permeability, and are considered as indicators of bacterial death (Benfield and Henriques, 2020). As expected, membrane permeabilization of K58A, K59N, and K58A/K59N double mutants was significantly reduced compared to that of WT Ang4 due to less binding. Interestingly, mutation of the K7 and K30 residues located near the center of the two N-terminal α-helices of Ang4 induced less permeability without affecting binding, indicating the involvement of these cationic residues in disrupting bacterial membranes. Indeed, pore formation is required to disrupt the membrane integrity of bacteria. It has been reported that the α-helix of AMPs contributes to the formation of pores by inserting helices into the lipid bilayer owing to its structural arrangement in which hydrophobic and hydrophilic faces are present in opposite directions (Zelezetsky and Tossi, 2006; Zhukovsky et al., 2019). In addition, studies on the structure-activity relationship of α-helical peptides revealed that conformational changes and flexibility of residues in the α-helix, are essential for the final pore formation or deeper insertion of molecules into the bilayer (Zelezetsky and Tossi, 2006). Thus, based on our findings, we propose that the initial association of Ang4 protein with the bacterial surface could be mediated through the cationic residues (K58 and K59) present in the cell binding segment, and that N-terminal two α-helices including cationic residues K7 and K30 are responsible for killing bacteria by increasing the permeability of bacterial membranes through the formation of pores.

It is known that the gut microbiota is influenced by antimicrobial proteins. However, to the best of our knowledge, no study has specifically explored alterations in microbial composition in Ang4-treated mice. Therefore, we performed studies to define the community structure of fecal bacteria in Ang4-treated mice. Our results demonstrated that, although there were no significant changes in alpha diversity, the observed features in Ang4-treated mice tended to be lower than those in PBS-treated mice. At the genus level, the results showed that the relative abundance of almost all bacteria that were significantly increased by the administration of Ang4 was considered beneficial for health. We found an increased abundance of *Lactobacillus*, which is regarded as protective bacteria and is thought to inhibit the proliferation of harmful bacteria by producing metabolites, such as lactic acid (Slover, 2008). In addition, *Akkermansia*, a mucin degrader that converts mucin to short chain fatty acids (SCFA) also increased in Ang4 treated mice, and is reported to influence immunoregulatory actions (Derrien et al., 2004). Furthermore, we observed an increased abundance of the symbiotic bacteria *Dubosiella*, equol-producing bacteria *Adlercreutzia*, and SCFA producer *Coriobacteriaceae* UCG-002 bacteria, which have been reported to possess many beneficial effects (Atkinson et al., 2005; Silva et al., 2020). However, certain SCFA-producing bacteria were decreased in Ang4-treated mice, such as *Roseburia*, which plays a significant role in regulating intestinal homeostasis through its metabolite, butyrate (Nie et al., 2021). In the Ang4-treated mice, we noted a significant reduction in the number of pathogenic bacteria, *Alistipes*, which is dominant in colorectal cancer (Parker et al., 2020). In addition, *Enterohabdus* bacteria, which have been reported to be associated with autoimmunity and IBDs (Clavel et al., 2009) were also significantly decreased. The observed changes in microbial composition induced by Ang4 suggest that the microbiota maintained by Ang4 may have anti-inflammatory potential.

It is reported that cationic antimicrobial proteins not only kill bacteria directly but also interact with host cells to modulate the inflammatory process (Hancock and Diamond, 2000; Hancock and Sahl, 2006). For direct killing, selectivity of certain AMPs are dependent on the composition of the bacterial membrane which is composed of distinct lipid structure in species of bacteria (Sohlenkamp and Geiger, 2015; Lei et al., 2021). On the other hand, AMPs exhibit immunomodulatory effects to clearance of bacteria or controlling inflammation through the recruitment and activation of innate immune cells such as neutrophils, monocytes, macrophage and dendritic cells, or proinflammatory cytokine suppression which could regulate gut microbiota (Hancock and Sahl, 2006; Mansour et al., 2014; Nazeer et al., 2021a). Thus, it is expected that Ang4 protein might interact with bacteria and host cells both to maintain intestinal bacteria composition. Indeed, extensive research is required to explore the interplay among Ang4 protein, host physiology and bacteria upon alteration of bacteria.

In conclusion, our study highlights that the antibacterial activity of Ang4 is achieved by the increase in bacterial membrane permeability, which is accomplished by the cationic residues present in the dual sites, including the N-terminal α-helix and cell-binding segment. This antibacterial function is distinct from that of conventional antibiotics, which inhibit cell wall synthesis, DNA replication, RNA transcription, and protein synthesis. Thus, Ang4 may not be susceptible to the rapid emergence of drug resistance, similar to that of antibiotics. In addition, alteration of the gut microflora by Ang4 reflects an increase in beneficial bacteria that are considered to have anti-inflammatory attributes. Because of its distinct bactericidal and potential anti-inflammatory properties, we believe that Ang4 could serve as a novel therapeutic agent to combat bacterial infections and inflammation.

## Data Availability Statement

The 16S rRNA gene sequencing datasets presented in this study can be found in the DDBJ online repositories under the accession number DRA013758. All data are included in the main text and Supplementary Material. Available at: https://ddbj.nig.ac.jp/public/ddbj_database/dra/fastq/DRA013/DRA013758/.

## Ethics Statement

The animal study was reviewed and approved by Chiba University Animal Care and Use Committee.

## Author Contributions

MFS and HK conceived the idea for this project and designed the experiments. MFS performed most of the experiments and analyzed the data. HA performed DNA isolation experiments on mice fecal bacteria and analyzed the 16S sequencing data. MS, FY, and KT provided technical support. WK generated the WT and H12A mutants. MFS, HA, and HK wrote the manuscript. All authors contributed to the article and approved the submitted version.

## Conflict of Interest

The authors declare that the research was conducted in the absence of any commercial or financial relationships that could be construed as a potential conflict of interest.

## Publisher’s Note

All claims expressed in this article are solely those of the authors and do not necessarily represent those of their affiliated organizations, or those of the publisher, the editors and the reviewers. Any product that may be evaluated in this article, or claim that may be made by its manufacturer, is not guaranteed or endorsed by the publisher.

## Footnotes

1
https://docs.qiime2.org/2021.11/

## Abbreviations

Ang4: angiogenin 4
hANG: human angiogenin
*S. typhimurium*: *Salmonella typhimurium*
AMPs: antimicrobial peptides
NPN: *N*-phenyl -1-naphthylamine
PI: propidium iodide
ELISA: enzyme-linked immunosorbent assay
NLS: nuclear localization sequence
LB: Luria broth
ASV: amplicon sequence variant
SCFA: short-chain fatty acids.
