# Supplementary material for: Identification of Crucial Amino Acid Residues for Antimicrobial Activity of Angiogenin 4 and Its Modulation of Gut Microbiota in Mice
Source: Front Microbiol. 2022 Jun 6;13:900948. doi: 10.3389/fmicb.2022.900948 (PMC9207454; doi:10.3389/fmicb.2022.900948)
Supplement: Supplementary file 1 [file Table_1.DOCX]

Supplementary Material

# Supplementary Figures and Tables

## Supplementary Figures


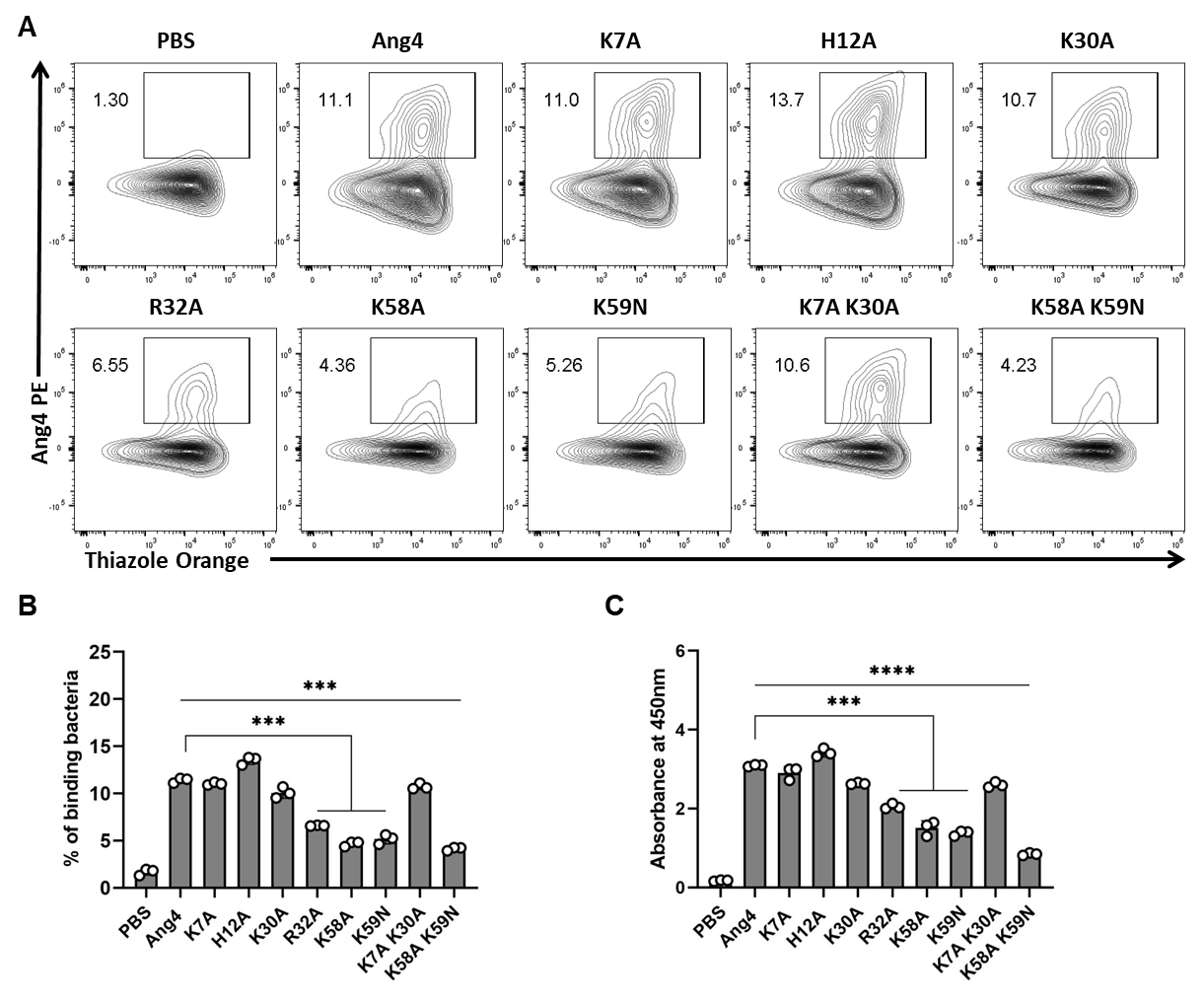


**Supplementary Figure 1.** **The binding of Ang4 and its mutants to bacteria from mouse feces**

**(A)** and **(B)** Flow cytometric analysis of the binding of Ang4 and its mutants to bacteria in mouse feces. PBS was used as a control. **(C)** Binding of Ang4 and its mutants to bacteria from mouse feces was evaluated by ELISA. All data are presented as mean ± SD from three independent experiments. P-values were determined by one-way ANOVA with Tukey's multiple comparisons test; ***P < 0.001, ****P<0.0001 vs. WT Ang4.

**
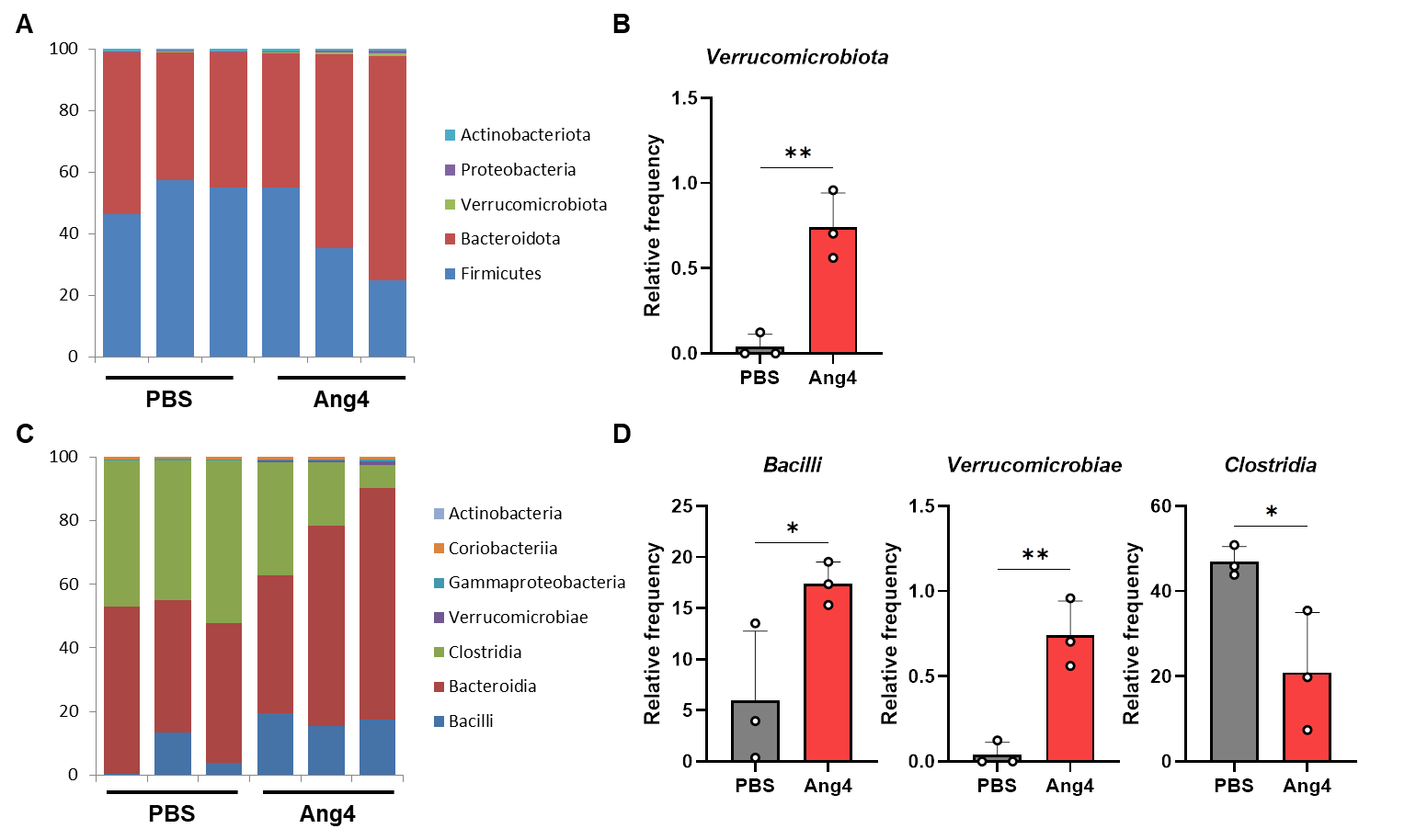
**

**Supplementary Figure 2. Taxonomical summarization of bacteria at different levels in Ang4- and PBS-treated mice**

**(A)** Bar plots show the average relative abundance of taxa at the phylum level. **(B)** Relative abundances of significantly altered bacteria at the phylum level. **(C)** Relative abundance of taxa at the class level. **(D)** Relative abundance of significantly altered bacteria at the class level. P-values were determined by two-tailed unpaired t-test; *P < 0.05, **P < 0.01.

## Supplementary Table

**Supplementary Table 1. Oligonucleotide primers and template DNA used for the generation of Ang4 mutants**

| Mutation | Template DNA |  | 5′→ 3′ |
| --- | --- | --- | --- |
| K7A | pNCMO2(Ang4) | Sense | CGAAGCATTCCTGCGCCAAC |
|  |  | Antisense | TAGCGTTCGTTTTGAGCAGCGAAAG |
| H12A | pNCMO2(Ang4) | Sense | CCAAGCATATGACGCTAAACC |
|  |  | Antisense | CGCAGGAATTTTTCGTAGCG |
| K30A | pNCMO2(Ang4) | Sense | GATGGCAGAACGCAAACTGACTTCC |
|  |  | Antisense | ATGGATTCGCAGTAGCGATCGTCG |
| R32A | pNCMO2(Ang4) | Sense | GAAGCAAAACTGACTTCCCCG |
|  |  | Antisense | TTTCATCATGGATTCGCAGTAGC |
| K58A | pNCMO2(Ang4) | Sense | GCGCAAAAGGCTCCCCATATGG |
|  |  | Antisense | CGCAGATAGCGCGGATGTTTTTC |
| K59N | pNCMO2(Ang4) | Sense | GCAAAAACGGCTCCCCATATGG |
|  |  | Antisense | CGCAGATAGCGCGGATGTTTTTC |
| K7A K30A | pNCMO2(K7A) | Sense | GATGGCAGAACGCAAACTGACTTCC |
|  |  | Antisense | ATGGATTCGCAGTAGCGATCGTCG |
| K58A K59N | pNCMO2(K59N) | Sense | GCGCAAACGGCTCCCCATAT |
|  |  | Antisense | CGCAGATAGCGCGGATGTTTTTC |
